# Neuropharmacological efficacy of metformin for stroke in rodents: A meta-analysis of preclinical trials

**DOI:** 10.3389/fphar.2022.1009169

**Published:** 2022-11-03

**Authors:** Wenqiao Fu, Yin Tang, Xudong Che, Jiahe Tan, Yinrui Ma, Zhaohui He

**Affiliations:** ^1^ Department of Neurosurgery, The First Affiliated Hospital of Chongqing Medical University, Chongqing, China; ^2^ Department of Anesthesiology, The First Affiliated Hospital of Chongqing Medical University, Chongqing, China

**Keywords:** metformin, stroke, neuropharmacology, animal models, meta-analysis

## Abstract

**Background:** Stroke, including ischemic stroke, intracerebral hemorrhage, and subarachnoid hemorrhage (SAH), remains a leading cause of mortality globally. Different stroke subtypes have similar detrimental effects in multiple fields of health. Previous research has shown that metformin plays a neuroprotective role in experimental animal models of stroke; however, a preclinical quantitative analysis on the ability of metformin to treat stroke is still lacking. This meta-analysis evaluates the efficacy of metformin in improving stroke prognosis in rodent models of stroke.

**Methods:** Relevant preclinical trials were retrieved from PubMed, EMBASE, and the Web of Science. The neurological score (NS), brain water content (BWC), infarct size, rotarod test, TUNEL, neuron quantity, microglia quantity, and p-AMPK levels were compared between a control group and a metformin group using the standardized mean difference (SMD) and corresponding confidence interval (CI). Quality was assessed with SYRCLE’s risk of bias tool.

**Results:** Fifteen articles published from 2010 to 2022 were included in the meta-analysis. The metformin group had statistically significant differences compared to the control group in the following aspects: NS (SMD −1.45; 95% CI −2.32, −0.58; *p* = 0.001), BWC (SMD −3.22; 95% CI −4.69, −1.76; *p* < 0.0001), infarct size (SMD −2.90; 95% CI −3.95, −1.85; *p* < 0.00001), rotarod test (SMD 2.55; 95% CI 1.87, 3.23; *p* < 0.00001), TUNEL (SMD -3.63; 95% CI −5.77, −1.48; *p* = 0.0009), neuron quantity (SMD 3.42; 95% CI 2.51, 4.34; *p* < 0.00001), microglia quantity (SMD −3.06; 95% CI -4.69, −1.44; *p* = 0.0002), and p-AMPK levels (SMD 2.92; 95% CI 2.02, 3.82; *p* < 0.00001). Furthermore, sensitivity analysis and stratified analysis were conducted for heterogeneous outcome indicators.

**Conclusion:** Overall, metformin treatment improves severe outcomes triggered by stroke. Despite the limitations intrinsic to animal studies, this systematic review may provide a vital reference for future high-quality preclinical trials and clinical use.

## Introduction

Cerebral stroke, a worldwide cerebrovascular disease (CVD) defined as arterial occlusion or rupture, initiates progressive and systemic pathophysiology, and results in functional impairment in multiple domains, such as motor, cognitive, and mental health (Sacco et al., 2013; Gu et al., 2021). Both ischemic and hemorrhagic stroke are characterized by oxidative stress, microglia polarization, neuroinflammation, and cell death, and ultimately lead to brain injury (Aronowski and Zhao, 2011; Hankey, 2014, 2017). People have long searched for effective therapeutic agents that promote recovery in stroke patients (Kuriakose and Xiao, 2020). However, most drugs proven to be therapeutic in preclinical studies have failed in clinical trials (Tao et al., 2020).

Metformin, derived from the plant *Galega officinalis*, has been widely used as a first-line treatment for type 2 diabetes (T2D) for over 60 years (Flory and Lipska, 2019). Many non-diabetic studies have reported that metformin delays tumor progression, slows aging, attenuates lung fibrosis, and reduces the risk of cardiovascular events (Rena et al., 2017; Farkhondeh et al., 2021). In recent years, metformin has been shown to exert a neuroprotective effect in middle cerebral artery occlusion (MCAO), ICH, and SAH animal models (Zeng et al., 2019; Lin et al., 2021; Zhang et al., 2022). Nevertheless, the overall efficacy of metformin in stroke prevention and recovery has been difficult to be evaluated due to methodological differences between studies.

To date, no systematic review or meta-analysis has been performed to synthesize the evidence from preclinical studies related to the effects of metformin in stroke or to assess their quality. Therefore, the purpose of this meta-analysis was to evaluate whether metformin could attenuate brain injury caused by stroke.

## Materials and methods

### Search strategy

A search was conducted by following the Preferred Reporting Items for Systematic Reviews and Meta-Analysis (PRISMA) guidelines (Moher et al., 2009). Preclinical trials related to the neuropharmacological efficacy of metformin for stroke in rodent models were retrieved from PubMed, EMBASE, and the Web of Science until 12 May 2022, with the publication language being restricted to English. The search was designed using the terms “[Metformin (Title/Abstract)] AND [Cerebral Hemorrhage (Title/Abstract) OR Ischemic Stroke (Title/Abstract) OR Subarachnoid Hemorrhage (Title/Abstract)],” as shown in Supplementary File S1.

### Inclusion and exclusion criteria

Articles were screened on the basis of the PICOS principle (population, intervention, control, outcome, and study design).

Research articles were included according to the following criteria: 1) stroke animal model, 2) includes a metformin experimental group, 3) includes a control group with placebo, 4) the effectiveness of metformin on stroke could be measured in the animal model, 5) findings were expressed or could be converted to the mean and standard deviation, and 6) other criteria: published in English.

Research articles were excluded according to the following criteria: 1) review articles, letters, and case reports; 2) repeated publications and abstracts without full text; 3) studies that did not report the number of animals in each group; and 4) animals with other diseases.

### Data collection

Data were extracted from a full-text article of each study by two reviewers (Fu and Tang), respectively, and any controversies were resolved by discussion with a third reviewer (Che). The following items were included: 1) the first author and publication year; 2) animal stroke models, including species, gender, age, and modeling; 3) anesthetic drugs and modality; 4) metformin administration time point and route, and metformin initial and total dosage; 5) time and outcome of measurement; and 6) methodological quality score. In the case that a study contained multiple experimental groups with different dosage and assessment times, each experimental group was independently considered as one comparison. The mean value and standard deviation were calculated statistically for continuous variables. GetData Graph Digitizer version 2.25 (http://getdata-graph-digitizer.com/) was used to obtain data from graphs. In addition, we calculated the standard deviation by multiplying the reported standard error of the mean (SEM) by the square root of the group.

### Quality assessment

SYRCLE’s risk of bias tool was used for quality assessment of all included studies by two independent reviewers (Fu and Tang) (Hooijmans et al., 2014). Disagreements were resolved by discussion with a third reviewer (Che).

### Statistical analysis

Outcomes included NS, BWC, infarct size, rotarod test, TUNEL, neuron quantity, microglia quantity, and p-AMPK levels. Review Manager (RevMan) 5.4 software (Cochrane Library, London, United Kingdom) and STATA 12.0 software (StataCorp, College Station, TX, United States) were used to analyze the data collected from the studies for meta-analysis. The SMD was used to describe the differences in the effect of metformin on stroke between the treatment group and the control group (Zeng et al., 2021). The heterogeneity among included studies was evaluated using the I-squared (I^2^) statistic. For I^2^ < 50% and I^2^ ≥ 50%, the fixed-effects models and the random-effects models were employed, respectively (DerSimonian and Laird, 1986; Vetter, 2019). Sensitivity analysis was used to identify the source of heterogeneity (Zeng et al., 2021). A stratified analysis was conducted to clarify the influence of the methodological differences, including animal model background, anesthetic agents and route, stroke model, metformin treatment time point, and measurement time. Publication bias was checked by a funnel plot, and asymmetry was estimated by Egger’s test and the trim-and-fill method (Egger et al., 1997). *p* < 0.05 was considered statistically significant.

## Results

### Study selection

**FIGURE 1:**
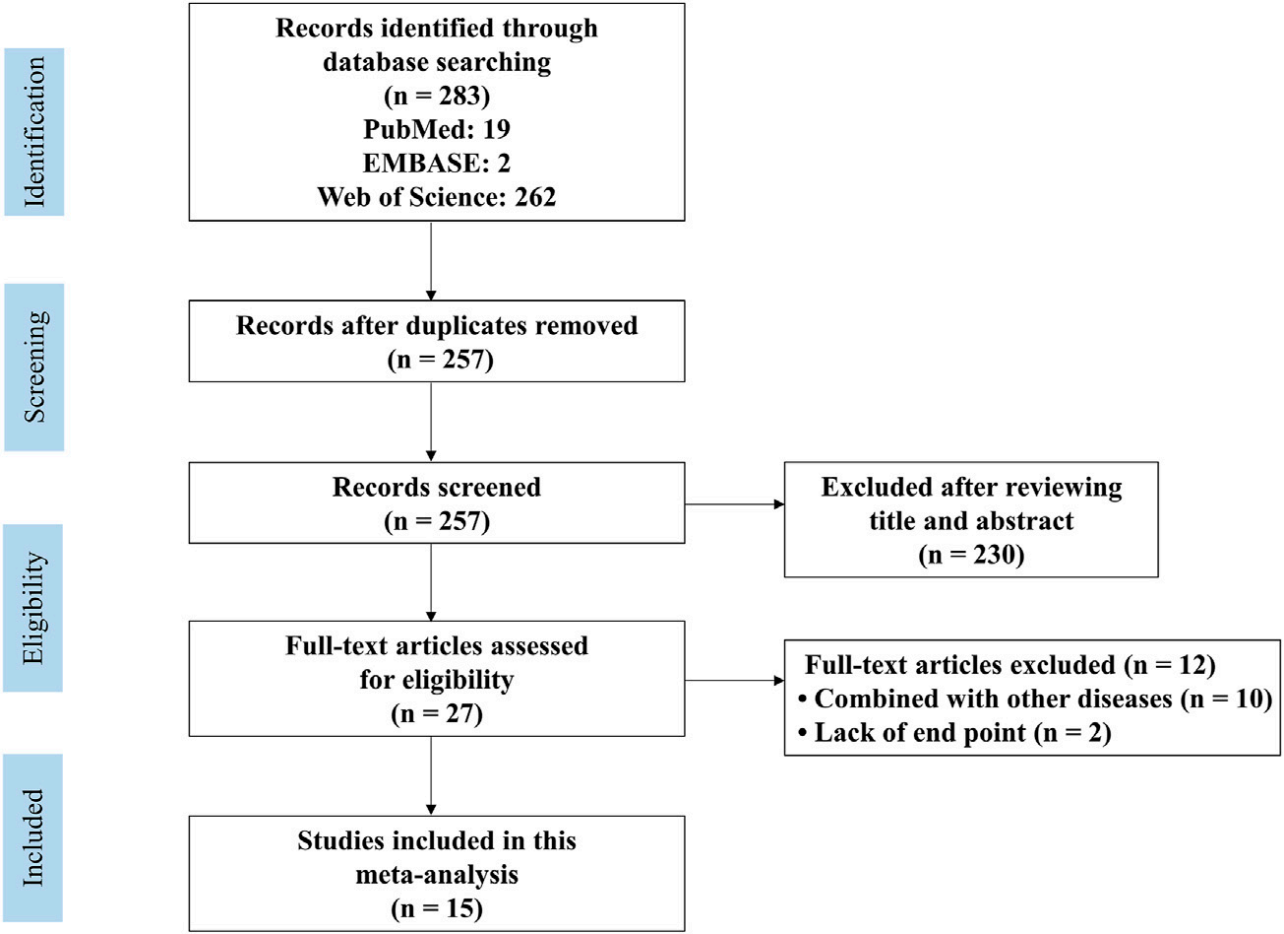
PRISMA flow diagram for review and selection process of studies included in meta-analysis of metformin in rodent models of stroke.

The flow diagram showing the screening process is presented in Figure 1. A total of 283 articles (19 in PubMed, two in EMBASE, and 262 in the Web of Science) were retrieved. Sixteen duplicate studies were removed. Subsequently, 230 studies were excluded due to lack of relevance. The full text of 27 studies was recorded. Twelve studies included animal models with comorbidities and were thus excluded. Finally, fifteen articles published from 2010 to 2022 were included (Li et al., 2010; Jin et al., 2014; Liu et al., 2014a; Liu et al., 2014b; Sarkaki et al., 2015; Zhu et al., 2015; Deng et al., 2016; Ge et al., 2017; Qi et al., 2017; Zeng et al., 2019; Lin et al., 2021; Zemgulyte et al., 2021; Jin et al., 2022; Liu et al., 2022; and Zhang et al., 2022).

### Study characteristics

The detailed characteristics of the included studies are presented in Table 1. All studies used adult male animals. The animal models were established in C57/BL6 mice (*n* = 5), Sprague–Dawley (SD) rats (*n* = 5), Wistar rats (*n* = 2), and CD-1 mice (*n* = 3). Anesthetic drugs included chloral hydrate (*n* = 5), sodium pentobarbital (*n* = 5), isoflurane (*n* = 3), and ketamine (*n* = 2). In addition to inhaling isoflurane, chloral hydrate, sodium pentobarbital, and ketamine were all administered by intraperitoneal injection (i.p.). Furthermore, MCAO (*n* = 11), ICH (*n* = 2), and SAH (*n* = 2) were chosen stroke models. The timing of metformin administration was divided into before MCAO (*n* = 3), after MCAO (*n* = 8), after SAH (*n* = 2), after ICH (*n* = 1), and before and after ICH (*n* = 1). Animals were subjected to i.p. (*n* = 10) and intragastric administration (i.g.) (*n* = 5) of metformin. The initial dosage of metformin was 10–200 mg/kg, and the most frequent dosage was 200 mg/kg (*n* = 6). The total dosage of metformin was 10–2,800 mg/kg.

**TABLE 1:** Characteristics of the included studies.

Author (year)	Animal, gender	Age	Anesthetic drug	Route	Model	Initial dosage	Total dosage	Treatment point	Route	Assessment time	Outcome measurement
Jin (2022)	C57/BL6 mice, male	Adult	Isoflurane	Inhalation	SAH	200 mg/kg	200 mg/kg	After SAH	i.p.	1 d	Neuron quantity, brain water content, TUNEL, p-AMPK, microglia quantity
Liu (2022)	SD rats, male	Adult	Sodium pentobarbital	i.p.	MCAO	10 mg/kg	10 mg/kg	After MCAO	i.p.	1 d	NS, brain water content, infarct size, TUNEL, p-AMPK, neuron quantity
Zhang (2022)	SD rats, male	Adult	Sodium pentobarbital	i.p.	SAH	20 mg/kg	20 mg/kg	After SAH	Intragastric administration	1 d	TUNEL, brain water content, p-AMPK
Lin (2021)	C57/BL6 mice, male	Adult	Sodium pentobarbital	i.p.	ICH	150 mg/kg	100 mg/kg	Before and after ICH	Intragastric administration	1 d	NS, rotarod test
							450 mg/kg			3 d	NS, rotarod test
							1,050 mg/kg			7 d	NS, rotarod test
							450 mg/kg			3 d	brain water content, p-AMPK, TUNEL
Zemgulyte (2021)	Wistar rats, male	Adult	Isoflurane	Inhalation	MCAO	50 mg/kg	100 mg/kg	After MCAO	i.p.	2 d	Neuron quantity, microglia quantity
							250 mg/kg			5 d	Infarct size
Zeng (2019)	C57/BL6 mice, male	Adult	Chloral hydrate	i.p.	MCAO	200 mg/kg	200 mg/kg	After MCAO	i.p.	1 d	NS, infarct size, TUNEL, neuron quantity, brain water content
							600 mg/kg			3 d	NS, infarct size, rotarod test
							1,400 mg/kg			7 d	NS, infarct size, rotarod test
							2,800 mg/kg			14 d	NS, infarct size, rotarod test
Ge (2017)	SD rats, male	Adult	Chloral hydrate	i.p.	MCAO	200 mg/kg	1,000 mg/kg	After MCAO	Intragastric administration	5 d	Neuron quantity
Qi (2017)	SD rats, male	Adult	Sodium pentobarbital	i.p.	ICH	100 mg/kg	100 mg/kg	After ICH	Intragastric administration	1 d	NS
							200 mg/kg			2 d	NS
							300 mg/kg			3 d	NS
							400 mg/kg			4 d	NS
							500 mg/kg			5 d	NS
							600 mg/kg			6 d	NS
							700 mg/kg			7 d	NS, neuron quantity, brain water content
Deng (2016)	C57/BL6 mice, male	Adult	Chloral hydrate	i.p.	MCAO	10 mg/kg	70 mg/kg	Before MCAO	i.p.	1 d	NS, infarct size
Zhu (2015)	SD rats, male	Adult	Chloral hydrate	i.p.	MCAO	50 mg/kg	1,050 mg/kg	Before MCAO	i.p.	1 d	NS, infarct size
										4 d	NS, infarct size
Sarkaki (2015)	Wistar rats, male	Adult	Chloral hydrate	i.p.	MCAO	200 mg/kg	600 mg/kg	After MCAO	Intragastric administration	3 d	NS
Jin (2014)	CD-1 mice, male	Adult	Isoflurane	Inhalation	MCAO	50 mg/kg	150 mg/kg	After MCAO	i.p.	3 d	p-AMPK
							700 mg/kg			14 d	p-AMPK
							2,550 mg/kg			14 d	Rotarod test
							1,050 mg/kg			21 d	Rotarod test
							1,400 mg/kg			28 d	Rotarod test
							650 mg/kg			13 d	NS
							1,350 mg/kg			27 d	NS
							1,450 mg/kg			29	NS
							1,500 mg/kg			30 d	infarct size
Liu (2014a)	CD-1 mice, male	Adult	Ketamine	i.p	MCAO	200 mg/kg	200 mg/kg	After MCAO	i.p	1 d	p-AMPK
Liu (2014b)	CD-1 mice, male	Adult	Ketamine	i.p	MCAO	200 mg/kg	200 mg/kg	After MCAO	i.p	1 d	infarct size
							600 mg/kg			3 d	NS, rotarod test, infarct size
							1,400 mg/kg			7 d	NS, rotarod test
							2,800 mg/kg			14 d	NS, rotarod test, p-AMPK
Li (2010)	C57/BL6 mice, male	Adult	Sodium pentobarbital	i.p	MCAO	50 mg/kg	1,050 mg/kg	Before MCAO	i.p	4 h	p-AMPK
										1 d	NS, infarct size

SD, Sprague–Dawley; MACO, middle cerebral artery occlusion; ICH, intracerebral hemorrhage; SAH, subarachnoid hemorrhage; i.p., intraperitoneal injection; h, hour; d, day; NS, neurological score.

### Quality assessment

The details of study quality are presented in Table 2. High and low scores represent high quality and low quality in the methodology, respectively. Most of the included studies scored between 2 to 8. All studies described analogous baseline characteristics, selecting outcome recording and free of counting sample sizes. Eleven studies reported the stochastic distribution. Two studies described the distribution concealment and blinding methods. Three studies described the blinded evaluation of results. In addition, there were seven studies that reported random collection of outcome measures, and there were three studies with other analysis bias.

**TABLE 2:** Methodological quality of studies.

Study (year)	1	2	3	4	5	6	7	8	9	10	Score
Jin (2022)	+	+	※	+	※	+	※	−	+	※	5
Liu (2022)	※	+	※	※	※	※	※	−	+	※	2
Zhang (2022)	+	+	+	+	+	+	+	−	+	※	8
Lin (2021)	※	+	※	※	※	※	※	−	+	※	2
Zemgulyte (2021)	※	+	※	※	※	※	※	−	+	※	2
Zeng (2019)	+	+	※	※	※	+	※	−	+	※	4
Ge (2017)	※	+	※	※	※	※	※	−	+	+	3
Qi (2017)	+	+	※	※	※	+	※	−	+	※	3
Deng (2016)	※	+	※	※	※	+	+	−	+	+	5
Zhu (2015)	※	+	※	※	※	※	※	−	+	※	2
Sarkaki (2015)	※	+	※	※	※	※	※	−	+	※	2
Jin (2014)	+	+	※	+	※	+	※	−	+	※	5
Liu (2014a)	※	+	※	※	※	※	※	−	+	※	2
Liu (2014b)	+	+	※	+	※	+	※	−	+	※	5
Li (2010)	※	+	+	※	+	※	+	−	+	+	6

1-stochastic distribution sequence; 2-analogous baseline traits; 3-distribution concealment; 4-stochastic housing; 5-blinded intervening; 6-random collection for outcome measurement; 7-blinded evaluation of result; 8-unfinished outcome data; 9-selecting outcome recording; 10-else sources of bias. +: yes; −: no; ※: unclear.

### Meta-analysis

**FIGURE 2:**
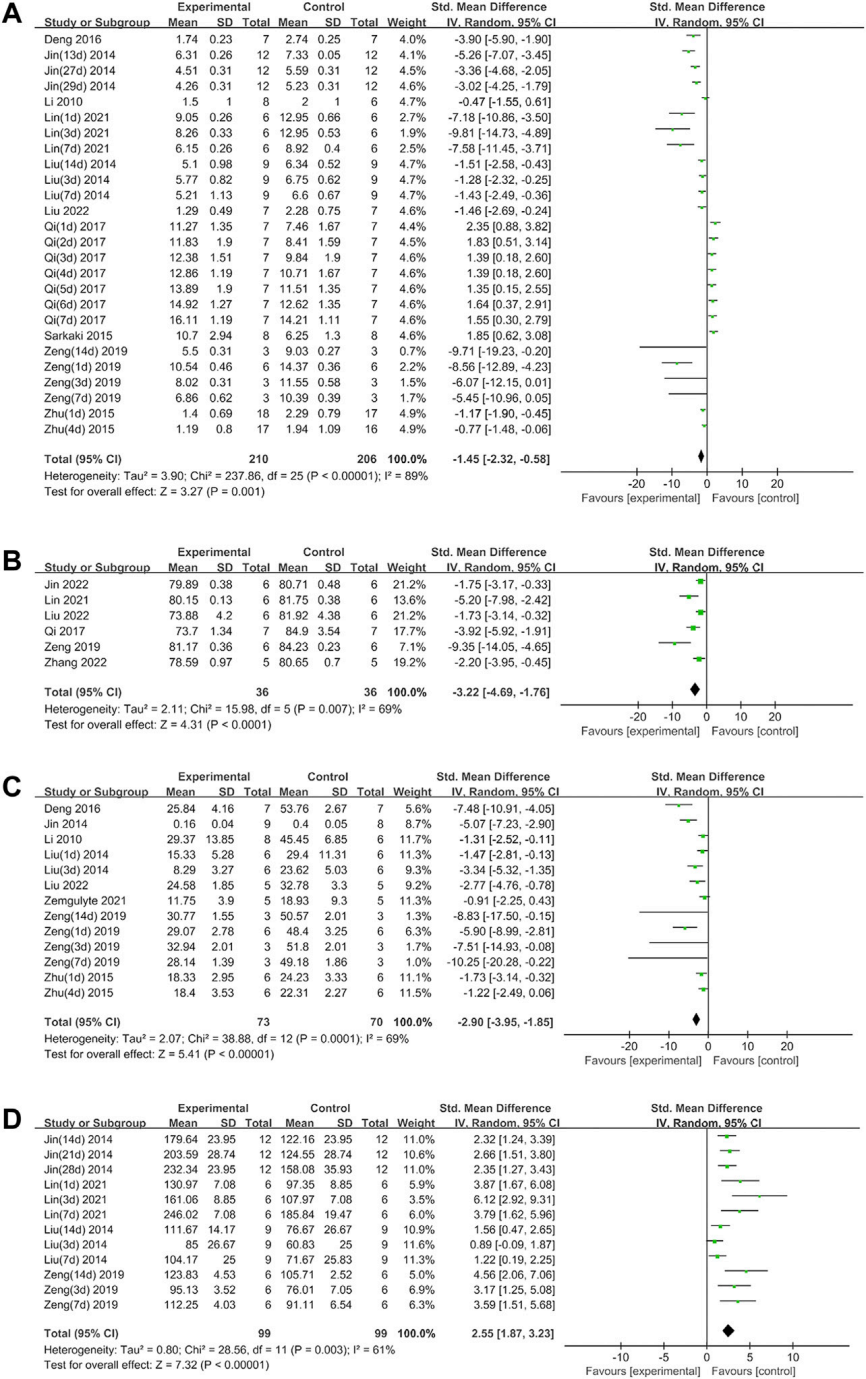
**(A)** Forest plot analyzing the effect of metformin treatment of NS. **(B)** Forest plot analyzing the effect of metformin treatment of the brain water content. **(C)** Forest plot analyzing the effect of metformin treatment of the infarct size. **(D)** Forest plot analyzing the effect of metformin treatment of the rotarod test.

**FIGURE 3:**
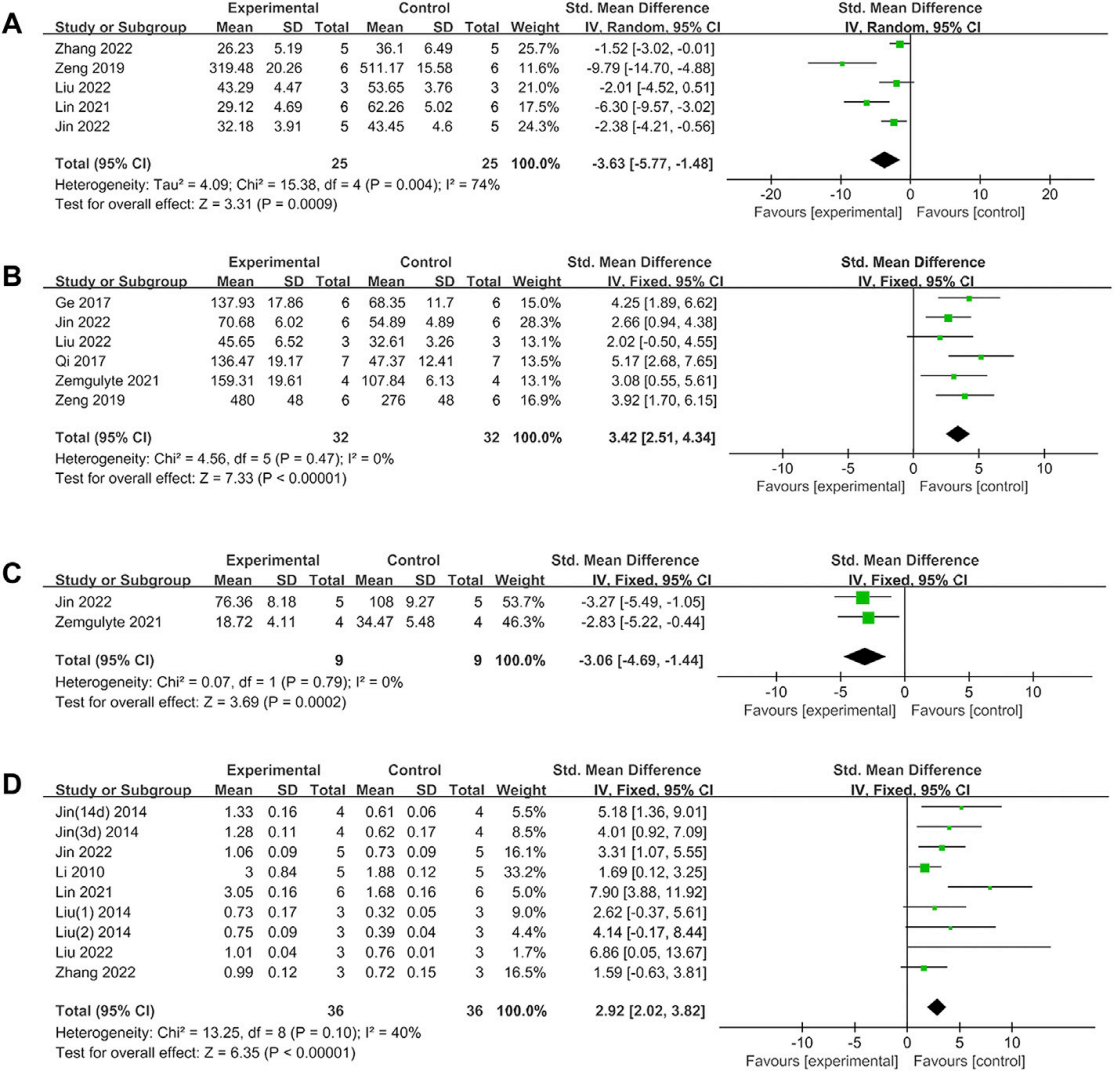
**(A)**Forest plot analyzing the effect of metformin treatment of TUNEL. **(B)** Forest plot analyzing the effect of metformin treatment of the neuron quantity. **(C)** Forest plot analyzing the effect of metformin treatment of the microglia quantity. **(D)** Forest plot analyzing the effect of metformin treatment of p-AMPK.

Metformin positively affected NS outcomes by an SMD of −1.45 (95% CI: −2.32, −0.58; *p* = 0.001, 10 studies, 26 comparisons; Figure 2A), with statistically significant heterogeneity (*I* ^2^ = 89%; *p* < 0.00001). Treatment with metformin reduced BWC by an SMD of −3.22 (95% CI: −4.69, −1.76; *p* < 0.0001, 6 studies; Figure 2B), with statistically significant heterogeneity (I^2^ = 69%; *p* = 0.007). Metformin also reduced the infarct size by an SMD of −2.90 (95% CI: −3.95, −1.85; *p* < 0.00001, 8 studies, 13 comparisons; Figure 2C), with statistically significant heterogeneity (I^2^ = 69%; *p* = 0.0001). Furthermore, metformin improved rotarod test results by an SMD of 2.55 (95% CI: 1.87, 3.23; *p* < 0.00001, 4 studies, 12 comparisons; Figure 2D), with statistically significant heterogeneity (I^2^ = 61%; *p* = 0.003). Metformin administration decreased TUNEL-positive cells by an SMD of -3.63 (95% CI: 5.77, −1.48; *p* = 0.0009, 5 studies; Figure 3A), with statistically significant heterogeneity (I^2^ = 74%; *p* = 0.004). Therefore, we further performed stratified analysis based on the animal model background, anesthetic drugs and route, stroke subtype, metformin administration time point and route, outcome measurement time for NS, BWC, infarct size, rotarod test, and TUNEL.

Metformin treatment increased the neuron quantity by an SMD of 3.42 (95% CI: 2.51, 4.34; *p* < 0.00001, 6 studies; Figure 3B), with a low heterogeneity (I^2^ = 0%; *p* = 0.47). Metformin treatment reduced the microglia quantity by an SMD of −3.06 (95% CI: 4.69, -1.44; *p* = 0.0002, 2 studies; Figure 3C), with a low heterogeneity (I^2^ = 0%; *p* = 0.79). Metformin increased p-AMPK levels by an SMD of 2.92 (95% CI: 2.02, 3.82; *p* < 0.00001, 8 studies, 9 comparisons; Figure 3D), with a low heterogeneity (*I* ^*2*^ = 40%; *p* = 0.1). Thus, further stratified analysis was not performed.

### Stratified analysis

For NS, significant differences among subgroups were found based on the animal model background (*p* = 0.00001), anesthetic drugs (*p* = 0.0004), anesthetic route (*p* = 0.0003), stroke subtype (*p* = 0.01), metformin administration timepoint (*p* = 0.00001), and outcome measurement time (*p* = 0.005). Among these, a clear difference was indicated in the therapeutic effect by the anesthetic route and outcome measurement time, and the effect size was greater with inhalation (SMD −3.72, 95% CI: −4.92, −2.53) and with outcomes measured at more than 7 days after treatment (SMD −3.31, 95% CI: −4.74, −1.88) (Table 3).

**TABLE 3:** Stratified meta-analysis of the NS.

Subgroup	Study	SMD (95% CI)	Heterogeneity test		p
			I^2^ (%)	p	
1. Animal					
C57/BL6 mice	9	−6.03 (−8.79, −3.26)	84	0.00001	
SD rats	10	0.75 (−0.16, 1.66)	86	0.00001	
Wistar rats	1	1.85 (0.62, 3.08)			
CD-1 mice	6	−2.51 (−3.55, −1.46)	78	0.0005	
					0.00001
2. Anesthetic					
Choral hydrate	8	−2.37 (−4.03, −0.71)	86	0.00001	
Isoflurane	3	−3.72 (−4.92, −2.53)	52	0.12	
Sodium pentobarbital ketamine	12	−0.24 (−1.44, 0.96)	87	0.00001	
	3	−1.40 (−2.01, −0.79)	0	0.00001	
					0.0004
3. Anesthesia route					
i.p.	23	−0.98 (−1.84, −0.13)	87	0.00001	
Inhalation	3	−3.72 (−4.92, −2.53)	52	0.12	
					0.0003
4. Model					
MCAO	16	−2.09 (−2.95, −1.24)	83	0.00001	
ICH	10	-0.07 (-1,42, 1.27)	86	0.00001	
					0.01
5. Treatment point					
Before MCAO	4	−1.23 (−2.11, −0.34)	69	0.02	
After MCAO	12	−2.59 (−3.85, −1.34)	85	0.00001	
Before and after ICH	3	−7.92 (−10.27, −5.58)	0	0.69	
After ICH	7	1.60 (1.12, 2.08)	0	0.96	
					0.00001
6. Assessment time, days					
≤7	21	−0.90 (−1.79, −0.00)	88	0.00001	
＞7	5	−3.31 (−4.74, −1.88)	74	0.004	
					0.005

For BWC, there was no significant difference in the estimated effect size among the animal model background (*p* = 0.24), anesthetic route (*p* = 0.09), or metformin administration timepoint (*p* = 0.08). Significant differences among subgroups were based on anesthetic drugs (*p* = 0.008) and the stroke subtype (*p* = 0.04) (Table 4).

**TABLE 4:** Stratified meta-analysis of the brain water content.

Subgroup	Study	SMD (95% CI)	Heterogeneity test		p
			I^2^ (%)	p	
1. Animal					
C57/BL6 mice	3	−4.93 (−8.88, −0.97)	84	0.002	
SD rats	3	−2.46 (−3.68, −1.24)	36	0.21	
					0.24
2. Anesthetic					
Choral hydrate	1	−9.35 (−14.05, -4.65)			
Isoflurane	1	−1.75 (−3.17, −0.33)			
Sodium pentobarbital	4	−2.97 (−4.39, −1.55)	55	0.0001	
					0.008
3. Anesthesia route					
i.p.	5	−3.73 (−5.57, −1.89)	72	0.007	
Inhalation	1	−1.75 (−3.17, −0.33)			
					0.09
4. Model					
MCAO	2	−5.20 (−12.63, 2.24)	89	0.002	
ICH	2	−4.36 (−5.98, −2.73)	0	0.46	
SAH	2	−1.93 (−3.03, −0.82)	0	0.69	
					0.04
5. Treatment point					
After SAH	2	−1.93 (−3.03, −0.82)	0	0.69	
After MCAO	2	−5.20 (−12,63, 2.24)	89	0.002	
Before and after ICH	1	−5.20 (−7.98, −2.42)			
After ICH	1	−3.92 (−5.92, −1.91)			
					0.08

For infarct size, no significant difference in the estimated effect size was observed among the anesthetic drugs (*p* = 0.27), anesthetic route (*p* = 1.00), and metformin administration time point (*p* = 0.27). Significant differences among subgroups were found based on the animal model background (*p* = 0.03) and outcome measurement time (*p* = 0.02). Among them, there was a clear difference in the therapeutic effect by the outcome measurement time. The effect size was greater in groups with outcomes measured at more than 7 days (SMD −5.29, 95% CI: −7.39, −3.19) (Table 5).

**TABLE 5:** Stratified meta-analysis of the infarct size.

Subgroup	Study	SMD (95% CI)	Heterogeneity test		p
			I^2^ (%)	p	
1. Animal					
C57/BL6 mice	6	−5.90 (−9.38, −2.41)	78	0.0004	
SD rats	3	−1.69 (−2.55, −0.84)	0	0.44	
Wistar rats	1	−0.91 (−2.25, 0.43)			
CD-1 mice	3	−3.16 (−5.28, −1.04)	76	0.02	
					0.03
2. Anesthetic					
Choral hydrate	7	−4.50 (−6.82, −2.18)	75	0.0006	
Isoflurane	2	−2.90 (−6.97, 1.17)	90	0.001	
Sodium pentobarbital ketamine	2	−1.82 (−3.17, −0.46)	33	0.22	
	2	−2.25 (−4.06, −0.45)	57	0.13	
					0.27
3. Anesthesia route					
i.p.	11	−2.90 (−4.03, −1.77)	65	0.001	
Inhalation	2	−2.90 (−6.97, 1.17)	90	0.001	
					1.00
4. Treatment point					
Before MCAO	4	−2.22 (−3.78, −0.65)	75	0.008	
After MCAO	9	−3.43 (−4.92, −1.95)	67	0.002	
					0.27
5. Assessment time, days					
≤7	11	−2.50 (−3.51, −1.50)	65	0.001	
＞7	2	−5.29 (−7.39, −3.19)	0	0.41	
					0.02

For rotarod tests, no significant differences in the estimated effect size among the anesthetic route (*p* = 0.57) and outcome measurement time (*p* = 0.47) were observed. Significant differences among subgroups were found based on the animal model background (*p* = 0.0001), anesthetic drugs (*p* = 0.0001), stroke subtype (*p* = 0.007), and metformin administration time point (*p* = 0.007). Among these, there was a clear difference in the therapeutic effect by the animal model background, stroke subtype, and metformin administration time point. The effect size was greater in C57/BL6 mice (SMD 3.92, 95% CI 3.00, 4.84), the ICH model (SMD 4.27, 95% CI 2.87, 5.66), and before and after ICH (SMD 4.27, 95% CI 2.87, 5.66) (Table 6).

**TABLE 6:** Stratified meta-analysis of the rotarod test.

Subgroup	Study	SMD (95% CI)	Heterogeneity test		p
			I^2^ (%)	p	
1. Animal					
C57/BL6 mice	6	3.92 (3.00, 4.84)	0	0.73	
CD-1 mice	6	1.80 (1.23, 2.37)	43	0.12	
					0.0001
2. Anesthetic					
Choral hydrate	3	3.65 (2.42, 4.88)	0	0.68	
Isoflurane	3	2.43 (1.80, 3.07)	0	0.90	
Sodium pentobarbital	3	4.27 (2.87, 5.66)	0	0.45	
ketamine	3	1.20 (0.61, 1.79)	0	0.67	
					0.0001
3. Anesthesia route					
i.p.	9	2.78 (1.76, 3.79)	71	0.0006	
Inhalation	3	2.43 (1.80, 3.07)	0	0.90	
					0.57
4. Model					
MCAO	9	2.16 (1.53, 2.80)	54	0.03	
ICH	3	4.27 (2.87, 5.66)	0	0.45	
					0.007
5. Treatment point					
After MCAO	9	2.16 (1.53, 2.80)	54	0.03	
Before and after ICH	3	4.27 (2.87, 5.66)	0	0.45	
					0.007
6. Assessment time, days					
≤7	7	2.86 (1.62, 4.11)	73	0.001	
＞7	5	2.35 (1.72, 2.98)	24	0.26	
					0.47

For TUNEL, no significant difference was seen in the estimated effect size among the animal model background (*p* = 0.07) and anesthetic route (*p* = 0.3). Significant differences among subgroups were found with stratification by anesthetic drugs (*p* = 0.02), stroke subtype (*p* = 0.03), and metformin administration time point (*p* = 0.03) (Table 7).

**TABLE 7:** Stratified meta-analysis of TUNEL.

Subgroup	Study	SMD (95% CI)	Heterogeneity test		*p*
			*I* ^*2*^ (%)	*p*	
1. Animal					
C57/BL6 mice	3	−5.69 (−9.84, −1.53)	80	0.006	
SD rats	2	−1.65 (−2.94, −0.36)	0	0.74	
					0.07
2. Anesthetic					
Choral hydrate	1	−9,79 (−14.70, −4.88)			
Isoflurane	1	−2.38 (−4.21, −0.56)			
Sodium pentobarbital	3	−2.94 (−5.47, −0.42)	71	0.03	
					0.02
3. Anesthesia route					
i.p.	4	−4.30 (−7.41, −1.19)	80	0.002	
Inhalation	1	−2.38 (−4.21, −0.56)			
					0.3
4. Model					
MCAO	2	−5.60 (−13.21, 2.01)	87	0.006	
ICH	1	−6.30 (−9.57, −3.02)			
SAH	2	−1.87 (−3.03, −0.71)	0	0.47	
					0.03
5. Treatment point					
After MCAO	2	−5.60 (−13.21, 2.01)	87	0.006	
Before and after ICH	1	−6.30 (−9.57, −3.02)			
After SAH	2	−1.87 (−3.03, −0.71)	0	0.47	
					0.03

### Sensitivity analysis

**FIGURE 4:**
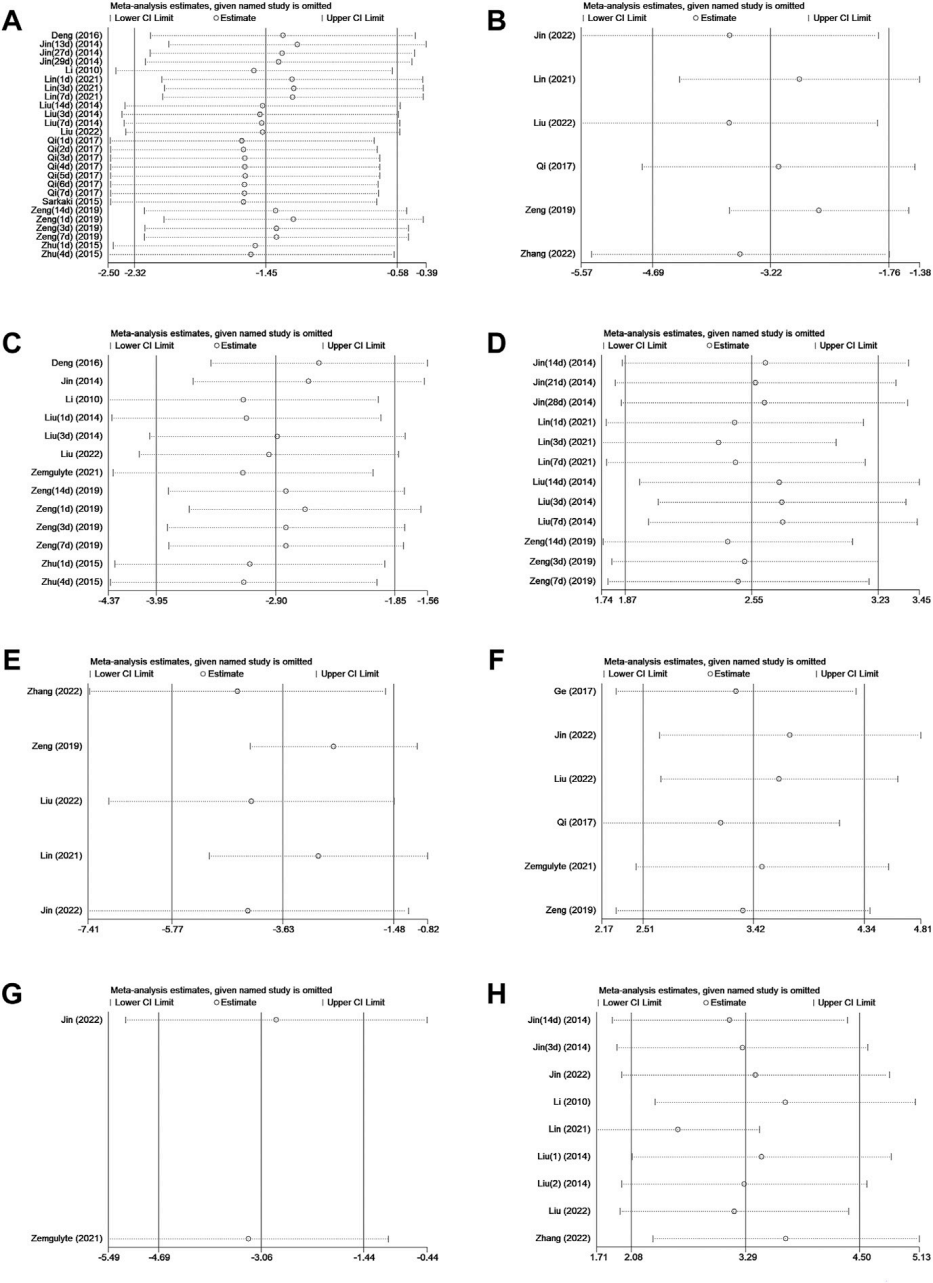
**(A)**Sensitivity analysis of metformin treatment of NS. **(B)** Sensitivity analysis of metformin treatment of the brain water content. **(C)** Sensitivity analysis of metformin treatment of the infarct size. **(D)** Sensitivity analysis of metformin treatment of the rotarod test. **(E)** Sensitivity analysis of metformin treatment of TUNEL. **(F)** Sensitivity analysis of metformin treatment of the neuron quantity. **(G)** Sensitivity analysis of metformin treatment of the microglia quantity. **(H)** Sensitivity analysis of metformin treatment of p-AMPK.

The robustness of our results was evaluated through a sensitivity analysis. The NS, BWC, infarct size, rotarod test, TUNEL, neuron quantity, microglia quantity, and p-AMPK levels were not significantly affected by any study for the pooled SMD (Figures 4A–H).

### Publication bias

**FIGURE 5:**
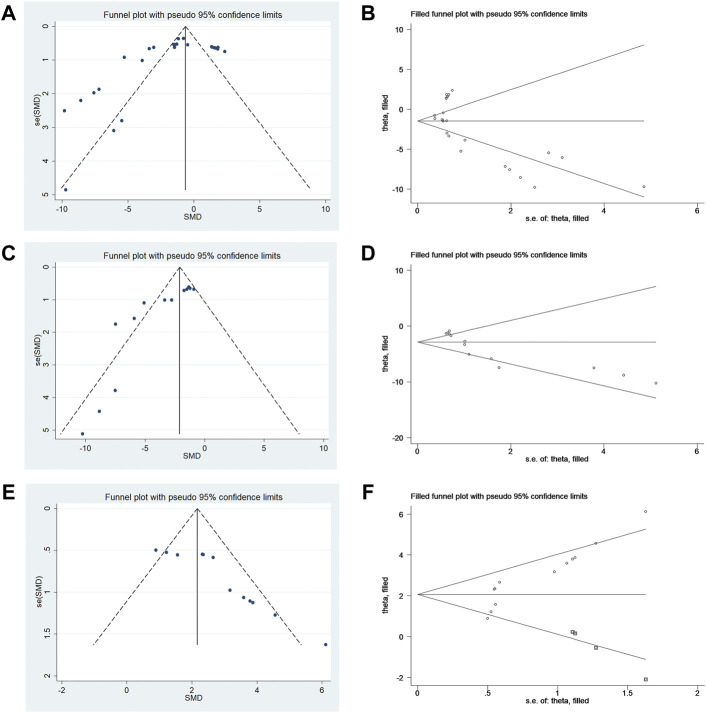
**(A)** Funnel plots for metformin treatment of NS. **(B)** Trim-and-fill analysis of metformin treatment of NS. **(C)** Funnel plots for metformin treatment of the infarct size. **(D)** Trim-and-fill analysis of metformin treatment of the infarct size. **(E)** Funnel plots for metformin treatment of the rotarod test. **(F)** Trim-and-fill analysis of metformin treatment of the rotarod test.

The publication bias analysis for outcome measures was performed with more than ten included articles. Conspicuous publication bias for the NS, infarct size, and rotarod test were suggested by visual inspection of the funnel plot (Figures 5A–C). Egger’s test confirmed the existence of publication bias in the NS (*p* = 0.036), infarct size % (*p* = 0.000), and rotarod test (*p* = 0.000). In this situation, the trim-and-fill analysis for NS, infarct size %, and rotarod test was conducted to estimate the results for the missing studies and recalculate effect estimates. The results of NS (SMD −1.451; 95% CI −2.321, −0.581; *p* = 0.000) and infarct size (SMD −2.901; 95% CI −3.951, −1.851; *p* = 0.000) were consistent, indicating no “missing” studies (Figures 5D,E). The result of the rotarod test (SMD 2.069; 95% CI 1.366, 2.772; *p* = 0.000) indicated that it had four “missing” studies (Figure 5F).

## Discussion

### Summary of evidence

To the best of our knowledge, no meta-analysis has been conducted to evaluate the neuropharmacological efficacy of metformin for stroke in rodents. The present study demonstrated that metformin has a neuropharmacological effect on stroke in animals. Overall, metformin significantly decreased BWC, infarct size, positive-TUNEL cells, and microglia quantity while increasing the neuron quantity and p-AMPK levels. Furthermore, metformin improved the NS and rotarod test. Collectively, the results of this meta-analysis of preclinical studies suggest that metformin may have a potential value as a therapeutic agent that protects against the detrimental effects of clinical stroke in patients.

### Potential mechanism for the effects of metformin

Stroke causes secondary pathological changes, including neuroinflammation, brain edema, and neurological function impairment. In addition, stroke decreases p-AMPK levels, which leads to neuronal loss and microglia polarization. The neuroprotective effects of increased p-AMPK have been shown in stroke studies (Lin et al., 2021; Liu et al., 2022; Zhang et al., 2022). Some researchers have found that metformin attenuates brain injury by activating the p-AMPK pathways and decreasing positive-TUNEL cells after stroke. NS, brain edema, and rotarod tests were significantly improved in a metformin treatment group compared with a stroke control group, suggesting that metformin treatment contributes to the recovery of neurological function in animals with stroke through anti-oxidative and anti-inflammation mechanisms (Li et al., 2010; Jin et al., 2014; Liu et al., 2014a; Liu et al., 2014b; Sarkaki et al., 2015; Zhu et al., 2015; Deng et al., 2016; Ge et al., 2017; Qi et al., 2017; Zeng et al., 2019; Lin et al., 2021; Zemgulyte et al., 2021; Jin et al., 2022; Liu et al., 2022; Zhang et al., 2022). Other research studies have reported that metformin could improve brain recovery through regulating microglia polarization (Jin et al., 2014b). These potential improvements in neurological function indicate that metformin could be a promising therapeutic and protective candidate for stroke in the future.

### Interpretation of stratified analysis

In this meta-analysis, metformin had significant neuropharmacological efficacy in reducing the BWC, infarct size, positive-TUNEL cells, and microglia quantity; in increasing the neuron quantity and p-AMPK levels; and in improving the NS and rotarod test. However, the heterogeneity of the data was statistically significant among NS, BWC, infarct size, rotarod test, and TUNEL. Thus, stratified analysis based on the animal model background, anesthetic drugs, anesthetic route, stroke subtype, metformin administration time point, and outcome measurement time was performed.

### Animal model background

Our stratified meta-analysis of NS, infarct size, and rotarod test found that the animal model background was a source of heterogeneity. The researchers selected C57/BL6 mice, SD rats, Wistar rats, and CD-1 mice for the study. Among them, C57/BL6 mice showed the largest effect size. Therefore, C57/BL6 mice may be the best rodent model to be used for the study of stroke.

### Anesthetic drugs and routes

To date, no systematic review has been conducted to discuss the effects of anesthetic use in rodent models of stroke. Our stratified meta-analysis of NS, BWC, rotarod test, and TUNEL found that anesthetic drugs were a source of heterogeneity. Researchers selected choral hydrate, isoflurane, sodium pentobarbital, and ketamine for this study. Among these, choral hydrate and sodium pentobarbital were chosen most often. Generally, the effect size of choral hydrate was larger than that of sodium pentobarbital, and chloral hydrate protected animals with ischemic stroke against brain infarct and edema, as described previously (Liu et al., 2015). In the present study, choral hydrate was found to be less widely used. In addition, studies have shown that chloral hydrate can have mutagenic and carcinogenic effects in animals (Maud et al., 2014). Thus, we cannot confirm whether choral hydrate is more suitable for the rodent models of stroke. Furthermore, our stratified meta-analysis of NS found that the anesthetic route was a source of heterogeneity, and inhalation has a larger effect size, while the number of studies that used i.p. was greater. Metformin efficacy might be overestimated due to a small number of studies. Therefore, we cannot confirm whether inhalation was the best choice of the anesthetic route. Overall, future studies need to focus on normalizing the selection of the anesthetic drug and route in stroke models.

### Stroke subtype

Our stratified meta-analysis of NS, BWC, rotarod test, and TUNEL found that the source of heterogeneity was related to the stroke subtype. The researchers selected MCAO, ICH, and SAH for analysis, and MCAO was the most used. In addition, NS and BWC, as the primary outcome measures of stroke, had the largest effect sizes when MACO was used. Therefore, metformin treatment may be more appropriate for the MCAO stroke subtype. In the future, more studies are needed to confirm this conclusion.

### Time point of metformin administration

Our stratified meta-analysis of NS, rotarod test, and TUNEL found that the time point of metformin administration was a source of heterogeneity. The researchers selected before MCAO, after MCAO, after SAH, before and after ICH, and after ICH for analysis. Among these, metformin administration before and after ICH had the largest effect, despite being used in the smallest number of studies. In the clinic, treatment is generally performed after a stroke has occurred. Therefore, future studies are needed to confirm the best time point for metformin administration for stroke treatment.

### Outcome measurement time

Our stratified meta-analysis of NS and infarct size found that outcome measurement time was a source of heterogeneity. The analyzed studies selected less than 7 days and more than 7 days as outcome measurement time points for the present analysis. Between the time points, more than 7 days had a larger effect size, although the number of the studies was smaller. Therefore, it is unclear which is the best time to measure outcomes, and more comparisons are needed. In fact, early detection of outcome indicators is favorable for assessing the severity of stroke and starting appropriate treatment. Similarly, long-term monitoring also helps in improving our understanding of the development of stroke, and it provides more references for formulating better treatment plans.

### Limitations

Several limitations exist in our study. Although we searched the vast majority of influential databases, there is still a shortage in the retrieved articles due to the fact that only articles published in English were included and some studies with negative results were ignored. Thus, the effect size of metformin may be exaggerated. The quality of this study was reduced due to some degree of heterogeneity across analyzed studies. The animal model background, anesthetic drugs and route, stroke subtype, metformin administration time point, and outcome measurement time may influence the quality. The differences among subgroups may not have been significant due to the lack of sufficient data for statistics, despite performing a stratified analysis. Therefore, more sufficient evidence is needed for statistical analysis in the future. In addition, varied intervention time spans may lead to substantial heterogeneity among the studies. Overall, significant work remains to be done for the clinical translation of metformin treatment for stroke patients.

### Future prospects

Although it has been suggested that patients with stroke on treatment with metformin have a better functional outcome in a few clinical research studies, there has been a lack of specific mechanism to prove that metformin could reduce brain damage caused by stroke (Cheng et al., 2014; Westphal et al., 2020; Kersten et al., 2022). In this review, we found metformin played a neuroprotective role through anti-oxidative and anti-inflammation mechanisms after stroke. Although this review was only conducted on preclinical studies, it reminds us that metformin may be one of the drugs used to treat stroke in the future.

## Conclusions

Our meta-analysis revealed that metformin treatment plays a neuroprotective role and improves pathological and behavioral outcomes in rodent models of stroke. Although multiple limitations regarding animal study methodology exist, the results of our study may provide an important reference for future preclinical and clinical studies on stroke outcome recovery.

## Data Availability

The original contributions presented in the study are included in the article/Supplementary Material; further inquiries can be directed to the corresponding author.
