# Supplementary material for: Neuropharmacological efficacy of metformin for stroke in rodents: A meta-analysis of preclinical trials
Source: Front Pharmacol. 2022 Nov 3;13:1009169. doi: 10.3389/fphar.2022.1009169 (PMC9669075; doi:10.3389/fphar.2022.1009169)
Supplement: Supplementary file 1 [file DataSheet1.docx]

**S1 Appendix. Search strategy**

**PubMed**

**#1** (("Cerebral Hemorrhage"[Mesh]) OR ("Ischemic Stroke"[Mesh]) OR ("Subarachnoid Hemorrhage"[Mesh]))

**#2** (((((((((((((((((((((((Hemorrhage, Cerebrum[Title/Abstract]) OR (Cerebrum Hemorrhage[Title/Abstract])) OR (Cerebrum Hemorrhages[Title/Abstract])) OR (Hemorrhages, Cerebrum[Title/Abstract])) OR (Cerebral Parenchymal Hemorrhage[Title/Abstract])) OR (Cerebral Parenchymal Hemorrhages[Title/Abstract])) OR (Hemorrhage, Cerebral Parenchymal[Title/Abstract])) OR (Hemorrhages, Cerebral Parenchymal[Title/Abstract])) OR (Parenchymal Hemorrhage, Cerebral[Title/Abstract])) OR (Parenchymal Hemorrhages, Cerebral[Title/Abstract])) OR (Intracerebral Hemorrhage[Title/Abstract])) OR (Hemorrhage, Intracerebral[Title/Abstract])) OR (Hemorrhages, Intracerebral[Title/Abstract])) OR (Intracerebral Hemorrhages[Title/Abstract])) OR (Hemorrhage, Cerebral[Title/Abstract])) OR (Cerebral Hemorrhages[Title/Abstract])) OR (Hemorrhages, Cerebral[Title/Abstract])) OR (Brain Hemorrhage, Cerebral[Title/Abstract])) OR (Brain Hemorrhages, Cerebral[Title/Abstract])) OR (Cerebral Brain Hemorrhage[Title/Abstract])) OR (Cerebral Brain Hemorrhages[Title/Abstract])) OR (Hemorrhage, Cerebral Brain[Title/Abstract])) OR (Hemorrhages, Cerebral Brain[Title/Abstract])) OR ((((((((((((((((((((((((Ischemic Strokes[Title/Abstract]) OR (Stroke, Ischemic[Title/Abstract])) OR (Ischaemic Stroke[Title/Abstract])) OR (Ischaemic Strokes[Title/Abstract])) OR (Stroke, Ischaemic[Title/Abstract])) OR (Cryptogenic Ischemic Stroke[Title/Abstract])) OR (Cryptogenic Ischemic Strokes[Title/Abstract])) OR (Ischemic Stroke, Cryptogenic[Title/Abstract])) OR (Stroke, Cryptogenic Ischemic[Title/Abstract])) OR (Cryptogenic Stroke[Title/Abstract])) OR (Cryptogenic Strokes[Title/Abstract])) OR (Stroke, Cryptogenic[Title/Abstract])) OR (Cryptogenic Embolism Stroke[Title/Abstract])) OR (Cryptogenic Embolism Strokes[Title/Abstract])) OR (Embolism Stroke, Cryptogenic[Title/Abstract])) OR (Stroke, Cryptogenic Embolism[Title/Abstract])) OR (Wake-up Stroke[Title/Abstract])) OR (Stroke, Wake-up[Title/Abstract])) OR (Wake up Stroke[Title/Abstract])) OR (Wake-up Strokes[Title/Abstract])) OR (Acute Ischemic Stroke[Title/Abstract])) OR (Acute Ischemic Strokes[Title/Abstract])) OR (Ischemic Stroke, Acute[Title/Abstract])) OR (Stroke, Acute Ischemic[Title/Abstract])) OR (((((((((((((((((((((((((((((SAH (Subarachnoid Hemorrhage[Title/Abstract])) OR (SAHs (Subarachnoid Hemorrhage[Title/Abstract]))) OR (Hemorrhage, Subarachnoid[Title/Abstract])) OR (Hemorrhages, Subarachnoid[Title/Abstract])) OR (Subarachnoid Hemorrhages[Title/Abstract])) OR (Subarachnoid Hemorrhage, Aneurysmal[Title/Abstract])) OR (Aneurysmal Subarachnoid Hemorrhage[Title/Abstract])) OR (Aneurysmal Subarachnoid Hemorrhages[Title/Abstract])) OR (Hemorrhage, Aneurysmal Subarachnoid[Title/Abstract])) OR (Hemorrhages, Aneurysmal Subarachnoid[Title/Abstract])) OR (Subarachnoid Hemorrhages, Aneurysmal[Title/Abstract])) OR (Subarachnoid Hemorrhage, Spontaneous[Title/Abstract])) OR (Hemorrhage, Spontaneous Subarachnoid[Title/Abstract])) OR (Hemorrhages, Spontaneous Subarachnoid[Title/Abstract])) OR (Spontaneous Subarachnoid Hemorrhage[Title/Abstract])) OR (Spontaneous Subarachnoid Hemorrhages[Title/Abstract])) OR (Subarachnoid Hemorrhages, Spontaneous[Title/Abstract])) OR (Perinatal Subarachnoid Hemorrhage[Title/Abstract])) OR (Hemorrhage, Perinatal Subarachnoid[Title/Abstract])) OR (Hemorrhages, Perinatal Subarachnoid[Title/Abstract])) OR (Perinatal Subarachnoid Hemorrhages[Title/Abstract])) OR (Subarachnoid Hemorrhage, Perinatal[Title/Abstract])) OR (Subarachnoid Hemorrhages, Perinatal[Title/Abstract])) OR (Subarachnoid Hemorrhage, Intracranial[Title/Abstract])) OR (Hemorrhage, Intracranial Subarachnoid[Title/Abstract])) OR (Hemorrhages, Intracranial Subarachnoid[Title/Abstract])) OR (Intracranial Subarachnoid Hemorrhage[Title/Abstract])) OR (Intracranial Subarachnoid Hemorrhages[Title/Abstract])) OR (Subarachnoid Hemorrhages, Intracranial[Title/Abstract]))

**#3**  #1 or #2

**#4** **("Metformin"[Mesh])**

**#5** **(((((((Dimethylbiguanidine[Title/Abstract]) OR (Dimethylguanylguanidine[Title/Abstract])) OR (Glucophage[Title/Abstract])) OR (Metformin Hydrochloride[Title/Abstract])) OR (Hydrochloride, Metformin[Title/Abstract])) OR (Metformin HCl[Title/Abstract])) OR (HCl, Metformin[Title/Abstract]))**

**#6**  #4 or #5

**#7**  #3 and #6

**EMBASE**

**#1**  'brain hemorrhage'/exp OR 'ischemic stroke'/exp OR 'subarachnoid hemorrhage'/exp

**#2** 'hemorrhage, cerebrum':ab,ti OR 'cerebrum hemorrhage':ab,ti OR 'cerebrum hemorrhages':ab,ti OR 'hemorrhages, cerebrum':ab,ti OR 'cerebral parenchymal hemorrhage':ab,ti OR 'cerebral parenchymal hemorrhages':ab,ti OR 'hemorrhage, cerebral parenchymal':ab,ti OR 'hemorrhages, cerebral parenchymal':ab,ti OR 'parenchymal hemorrhage, cerebral':ab,ti OR 'parenchymal hemorrhages, cerebral':ab,ti OR 'intracerebral hemorrhage':ab,ti OR 'hemorrhage, intracerebral':ab,ti OR 'hemorrhages, intracerebral':ab,ti OR 'intracerebral hemorrhages':ab,ti OR 'hemorrhage, cerebral':ab,ti OR 'cerebral hemorrhages':ab,ti OR 'hemorrhages, cerebral':ab,ti OR 'brain hemorrhage, cerebral':ab,ti OR 'brain hemorrhages, cerebral':ab,ti OR 'cerebral brain hemorrhage':ab,ti OR 'cerebral brain hemorrhages':ab,ti OR 'hemorrhage, cerebral brain':ab,ti OR 'hemorrhages, cerebral brain':ab,ti OR 'ischemic strokes':ab,ti OR 'stroke, ischemic':ab,ti OR 'ischaemic strokes':ab,ti OR 'ischaemic stroke':ab,ti OR 'stroke, ischaemic':ab,ti OR 'cryptogenic ischemic stroke':ab,ti OR 'cryptogenic ischemic strokes':ab,ti OR 'ischemic stroke, cryptogenic':ab,ti OR 'stroke, cryptogenic ischemic':ab,ti OR 'cryptogenic stroke':ab,ti OR 'cryptogenic strokes':ab,ti OR 'stroke, cryptogenic':ab,ti OR 'cryptogenic embolism stroke':ab,ti OR 'cryptogenic embolism strokes':ab,ti OR 'embolism stroke, cryptogenic':ab,ti OR 'stroke, cryptogenic embolism':ab,ti OR 'wake-up stroke':ab,ti OR 'stroke, wake-up':ab,ti OR 'wake up stroke':ab,ti OR 'wake-up strokes':ab,ti OR 'acute ischemic stroke':ab,ti OR 'acute ischemic strokes':ab,ti OR 'ischemic stroke, acute':ab,ti OR 'stroke, acute ischemic':ab,ti OR 'sah (subarachnoid hemorrhage)':ab,ti OR 'sahs (subarachnoid hemorrhage)':ab,ti OR 'hemorrhage, subarachnoid':ab,ti OR 'hemorrhages, subarachnoid':ab,ti OR 'subarachnoid hemorrhages':ab,ti OR 'subarachnoid hemorrhage, aneurysmal':ab,ti OR 'aneurysmal subarachnoid hemorrhage':ab,ti OR 'aneurysmal subarachnoid hemorrhages':ab,ti OR 'hemorrhage, aneurysmal subarachnoid':ab,ti OR 'hemorrhages, aneurysmal subarachnoid':ab,ti OR 'subarachnoid hemorrhages, aneurysmal':ab,ti OR 'subarachnoid hemorrhage, spontaneous':ab,ti OR 'hemorrhage, spontaneous subarachnoid':ab,ti OR 'hemorrhages, spontaneous subarachnoid':ab,ti OR 'spontaneous subarachnoid hemorrhage':ab,ti OR 'spontaneous subarachnoid hemorrhages':ab,ti OR 'subarachnoid hemorrhages, spontaneous':ab,ti OR 'perinatal subarachnoid hemorrhage':ab,ti OR 'hemorrhage, perinatal subarachnoid':ab,ti OR 'hemorrhages, perinatal subarachnoid':ab,ti OR 'perinatal subarachnoid hemorrhages':ab,ti OR 'subarachnoid hemorrhage, perinatal':ab,ti OR 'subarachnoid hemorrhages, perinatal':ab,ti OR 'subarachnoid hemorrhage, intracranial':ab,ti OR 'hemorrhage, intracranial subarachnoid':ab,ti OR 'hemorrhages, intracranial subarachnoid':ab,ti OR 'intracranial subarachnoid hemorrhage':ab,ti OR 'intracranial subarachnoid hemorrhages':ab,ti OR 'subarachnoid hemorrhages, intracranial':ab,ti

**#3**  #1 or #2

**#4** 'metformin'/exp

**#5**  **'dimethylbiguanidine':ab,ti OR 'dimethylguanylguanidine':ab,ti OR 'metformin':ab,ti OR 'metformin hydrochloride':ab,ti OR 'hydrochloride, metformin':ab,ti OR 'metformin hcl':ab,ti OR 'hcl, metformin':ab,ti**

**#6**  #4 or #5

**#7**  #3 and #6

**Web of Science**

**#1** TS= (Cerebral Hemorrhage) OR TS= (Ischemic Stroke) OR TS= (Subarachnoid Hemorrhage)

**#2** AB=( Hemorrhage, Cerebrum OR Cerebrum Hemorrhage OR Cerebrum Hemorrhages OR Hemorrhages, Cerebrum OR Cerebral Parenchymal Hemorrhage OR Cerebral Parenchymal Hemorrhages OR Hemorrhage, Cerebral Parenchymal OR Hemorrhages, Cerebral Parenchymal OR Parenchymal Hemorrhage, Cerebral OR Parenchymal Hemorrhages, Cerebral OR Intracerebral Hemorrhage OR Hemorrhage, Intracerebral OR Hemorrhages, Intracerebral OR Intracerebral Hemorrhages OR Hemorrhage, Cerebral OR Cerebral Hemorrhages OR Hemorrhages, Cerebral OR Brain Hemorrhage, Cerebral OR Brain Hemorrhages, Cerebral OR Cerebral Brain Hemorrhage OR Cerebral Brain Hemorrhages OR Hemorrhage, Cerebral Brain OR Hemorrhages, Cerebral Brain OR Ischemic Strokes OR Stroke, Ischemic OR Ischaemic Stroke OR Ischaemic Strokes OR Stroke, Ischaemic OR Cryptogenic Ischemic Stroke OR Cryptogenic Ischemic Strokes OR Ischemic Stroke, Cryptogenic OR Stroke, Cryptogenic Ischemic OR Cryptogenic Stroke OR Cryptogenic Strokes OR Stroke, Cryptogenic OR Cryptogenic Embolism Stroke OR Cryptogenic Embolism Strokes OR Embolism Stroke, Cryptogenic OR Stroke, Cryptogenic Embolism OR Wake-up Stroke OR Stroke, Wake-up OR Wake up Stroke OR Wake-up Strokes OR Acute Ischemic Stroke OR Acute Ischemic Strokes OR Ischemic Stroke, Acute OR Stroke, Acute Ischemic OR SAH Subarachnoid Hemorrhage OR SAHs Subarachnoid Hemorrhage OR Hemorrhage, Subarachnoid OR Hemorrhages, Subarachnoid OR Subarachnoid Hemorrhages OR Subarachnoid Hemorrhage, Aneurysmal OR Aneurysmal Subarachnoid Hemorrhage OR Aneurysmal Subarachnoid Hemorrhages OR Hemorrhage, Aneurysmal Subarachnoid OR Hemorrhages, Aneurysmal Subarachnoid OR Subarachnoid Hemorrhages, Aneurysmal OR Subarachnoid Hemorrhage, Spontaneous OR Hemorrhage, Spontaneous Subarachnoid OR Hemorrhages, Spontaneous Subarachnoid OR Spontaneous Subarachnoid Hemorrhage OR Spontaneous Subarachnoid Hemorrhages OR Subarachnoid Hemorrhages, Spontaneous OR Perinatal Subarachnoid Hemorrhage OR Hemorrhage, Perinatal Subarachnoid OR Hemorrhages, Perinatal Subarachnoid OR Perinatal Subarachnoid Hemorrhages OR Subarachnoid Hemorrhage, Perinatal OR Subarachnoid Hemorrhages, Perinatal OR Subarachnoid Hemorrhage, Intracranial OR Hemorrhage, Intracranial Subarachnoid OR Hemorrhages, Intracranial Subarachnoid OR Intracranial Subarachnoid Hemorrhage OR Intracranial Subarachnoid Hemorrhages OR Subarachnoid Hemorrhages, Intracranial)

**#3**  #1 or #2

**#4** **TS=((Metformin)**

**#5** **AB= (Dimethylbiguanidine OR Dimethylguanylguanidine OR Glucophage OR Metformin Hydrochloride OR Hydrochloride, Metformin OR Metformin HCl OR HCl, Metformin)**

**#6**  #4 or #5

**#7**  #3 and #6
